# Supplementary material for: Serum from COVID-19 patients early in the pandemic shows limited evidence of cross-neutralization against variants of concern
Source: bioRxiv. 2021 Nov 12:2021.11.10.468174. Preprint. [Version 1] doi: 10.1101/2021.11.10.468174 (PMC8597881; doi:10.1101/2021.11.10.468174)
Supplement: 1 [file NIHPP2021.11.10.468174V1-supplement-1.pdf]

bioRxiv preprint doi: https://doi.org/10.1101/2021.11.10.468174; this version posted November 12, 2021. The copyright holder for this preprint (which was not certified by peer review) is the author/funder. This article is a US Government work. It is not subject to copyright under 17 USC 105 and is also made available for use under a CC0 license.

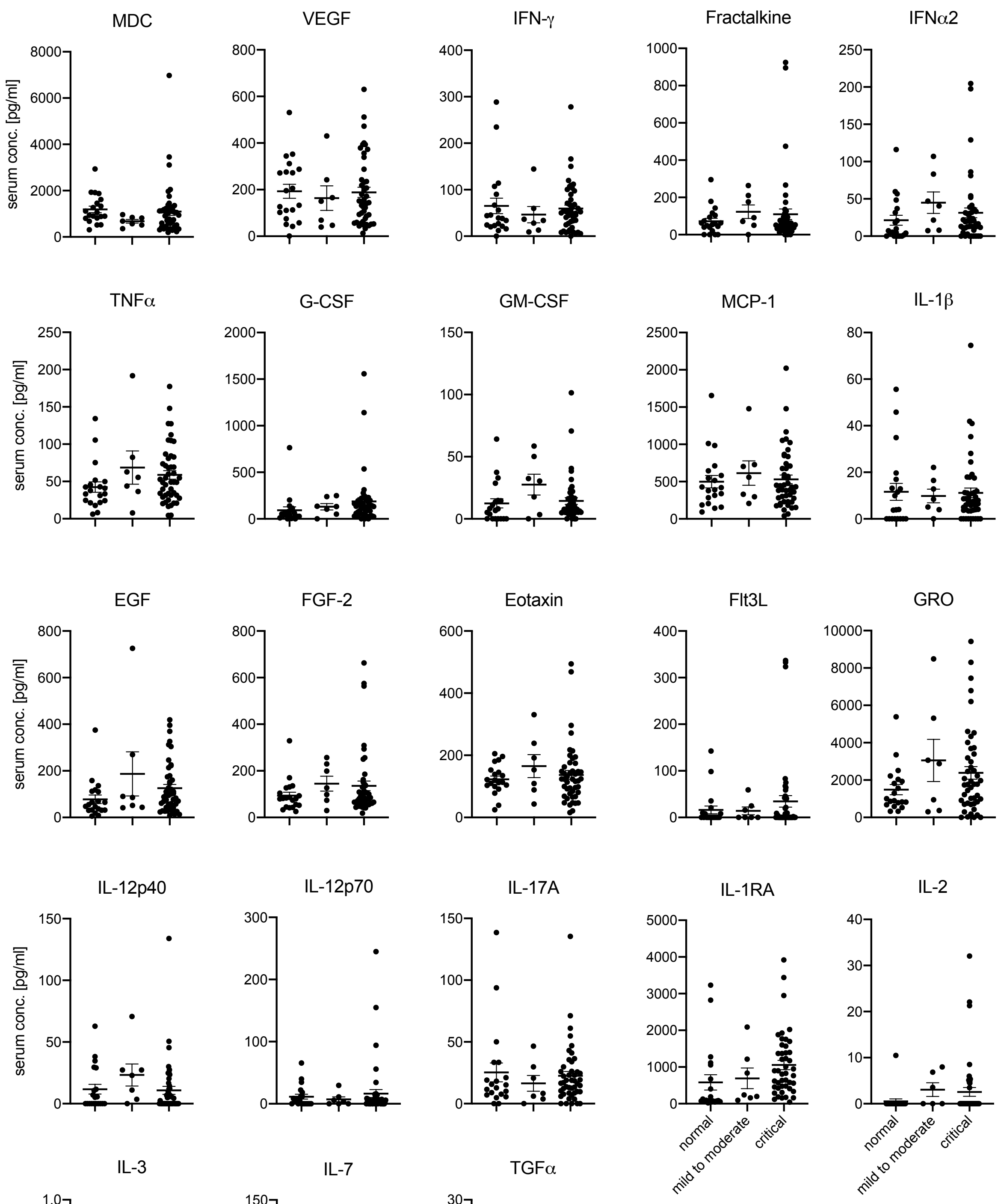

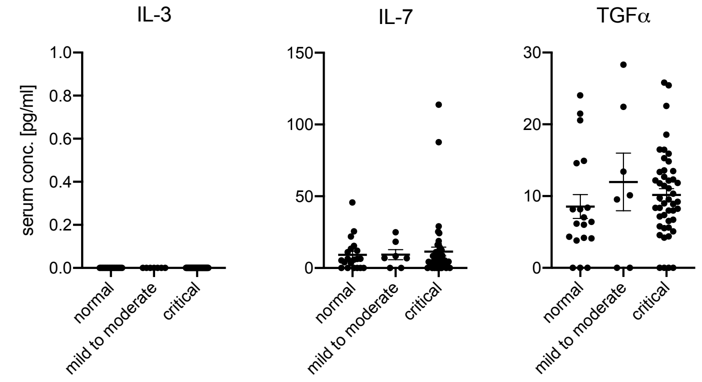

Supplementary Figure 1. Cytokine and chemokine levels for day-of-admission COVID-19 serum samples alongside 20 normal samples. Error bars represent standard error.

bioRxiv preprint doi: https://doi.org/10.1101/2021.11.10.468174; this version posted November 12, 2021. The copyright holder for this preprint (which was not certified by peer review) is the author/funder. This article is a US Government work. It is not subject to copyright under 17 USC 105 and is also made available for use under a CC0 license.

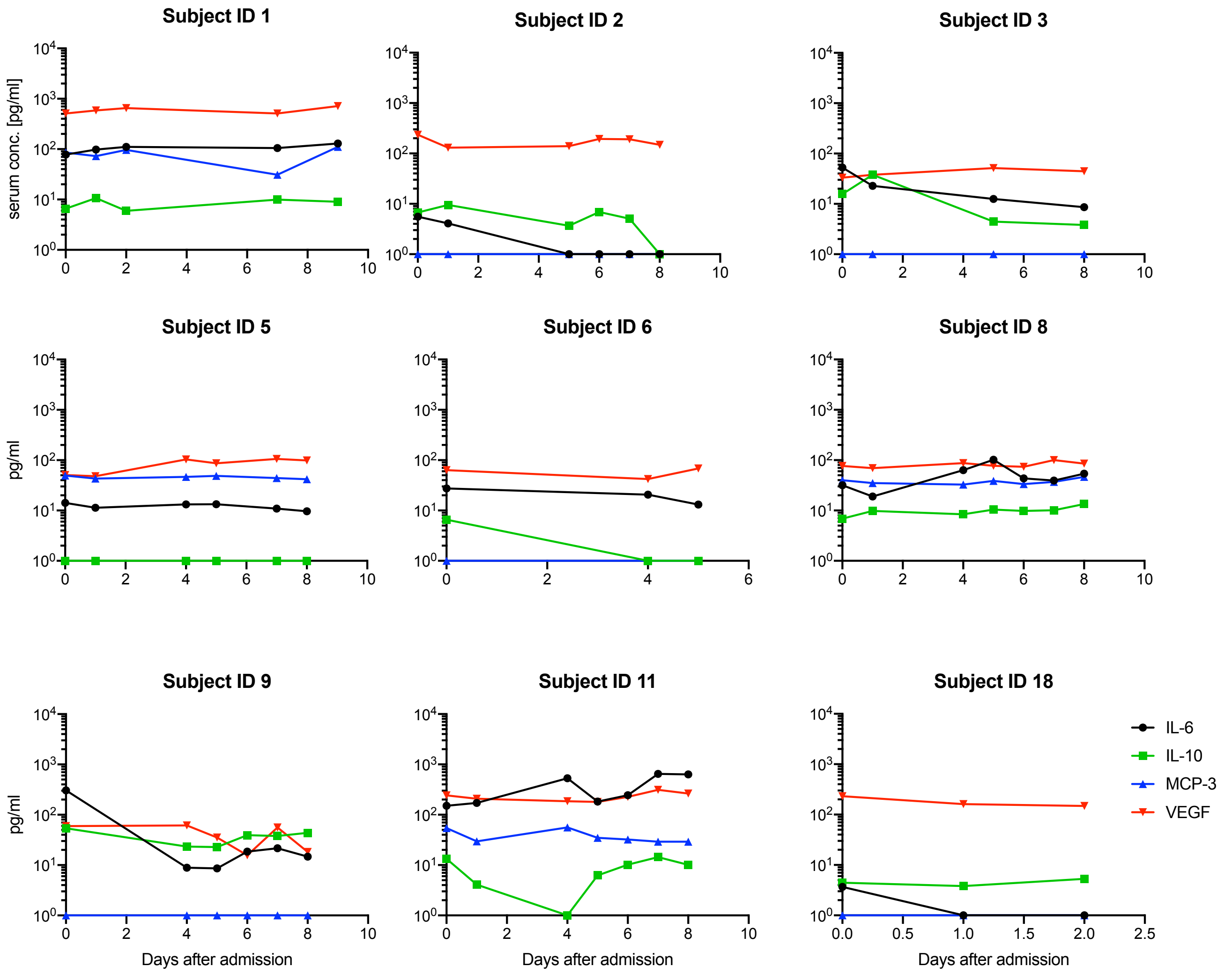

Supplementary Figure 2. Cytokine and chemokine levels of selected patients over time.

bioRxiv preprint doi: https://doi.org/10.1101/2021.11.10.468174; this version posted November 12, 2021. The copyright holder for this preprint (which was not certified by peer review) is the author/funder. This article is a US Government work. It is not subject to copyright under 17 USC 105 and is also made available for use under a CC0 license.

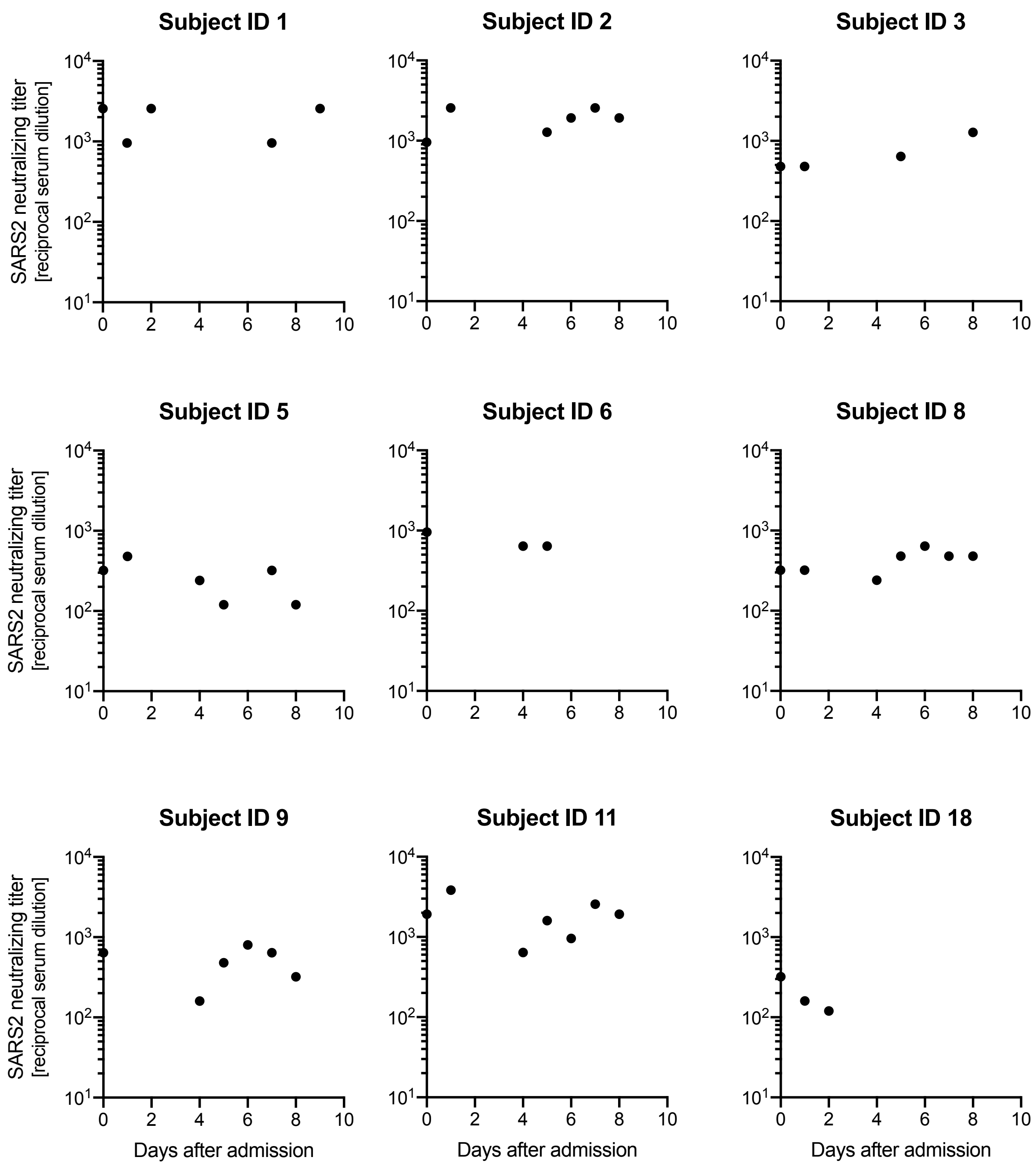

Supplementary Figure 3. Anti-SARS-CoV-2 neutralizing antibodies remain steady over time in selected patients.
